# Chili Pepper Jojutla Morelos (*Capsicum annuum* L.), CJ-2018: A Variety Resistant to *Bactericera cockerelli*

**DOI:** 10.3390/insects13080742

**Published:** 2022-08-18

**Authors:** Manuel Silva-Valenzuela, Reyna Isabel Rojas-Martínez, Victor M. Zúñiga-Mayo

**Affiliations:** 1 Postgrado en Fitosanidad-Fitopatología, Colegio de Postgraduados (CP), Campus Montecillo, km 36.5 Carretera México-Texcoco, Montecillo 56230, Estado de México, Mexico; 2 CONACyT, Postgrado en Fitosanidad-Fitopatología, Colegio de Postgraduados (CP), Campus Montecillo, km 36.5 Carretera México-Texcoco, Montecillo 56230, Estado de México, Mexico

**Keywords:** antixenosis, antibiosis, ‘Criollo de Jojutla’, resistance

## Abstract

**Simple Summary:**

*Bactericera cockerelli* is a pest in different crops including chili, one of the main vegetables worldwide. The presence of this pest in crops causes significant damage to their yield. The control of *B. cockerelli* is mainly conducted using insecticides, which causes a detrimental effect on the environment and favors the development of resistant pest populations. The identification of pest-resistant plant varieties is the first step towards their future incorporation into management programs. However, chili pepper varieties resistant to *B. cockerelli* have not been identified. In the present research, different parameters were evaluated in two chili varieties to find out if they are resistant to *B. cockerelli*. The results obtained showed that *B. cockerelli* laid fewer eggs in ‘Criollo de Jojutla’ (CJ-2018) compared to the control and that very few eggs managed to reach the adult stage, indicating that CJ-2018 was highly resistant to *B. cockerelli*. This work constitutes the first step towards the use of CJ-2018-resistant traits for the management of *B. cockerelli*.

**Abstract:**

Chili pepper is a vegetable of worldwide economic and gastronomic importance. The psyllid, *Bactericera cockerelli*, is an economically important pest in this crop, causing considerable losses in its production. Currently, the application of insecticides is the main way to control *B. cockerelli*. However, the use of varieties resistant to this insect is a viable alternative for its control and management. In this work, the oviposition rate, development, and survival of *B. cockerelli* in two native varieties of chili were evaluated. Choice and non-choice trials showed that the *B. cockerelli* oviposition was reduced on CJ-2018 by 92.17 and 80.18%, respectively, compared to the control. In CM-334, the insect showed a behavior similar to the control in the non-choice test, while in the choice test it laid more eggs on CM-334 compared to the control. The development and survival assay showed that only 1.33% of the eggs managed to reach the adult stage on CJ-2018. In contrast, on CM-334 the survival of *B. cockerelli* was similar to the control. These results suggest that CJ-2018 presented a resistance based on antixenosis and antibiosis against *B. cockerelli*.

## 1. Introduction

Chili pepper (*Capsicum annuum* L.) is one of the most produced vegetables in the world. In the last 15 years, the average area devoted to this crop was 124,289.40 ha [1]. In Mexico, chili cultivation holds great economic importance. During 2020, 3324 t of chili were produced, with an estimated value of $34,012 million pesos [1]. The main producing states are Chihuahua, Sinaloa, and Zacatecas. In addition, this vegetable is essential for Mexican cuisine; therefore, the national demand is high [1].

Among the biotic factors that limit chili pepper production, there are different diseases and pests, such as the psyllid *Bactericera cockerelli* (Sulc) (Hemiptera: Triozidae), which also affects potato and tomato crops [2,3,4]. In chili pepper, this insect causes direct damage by feeding on the plant and indirect damage when it transmits *Candidatus* Liberibacter solanacearum (*Ca*Lso), the causal agent of chili variegation, which reduces its production by up to 100%. Therefore, it is necessary to keep the population of *B. cockerelli* low [4,5]. Currently, control of *B. cockerelli* is conducted using insecticides. However, this exerts a selection pressure, which favors the reproduction of populations resistant to one or more active ingredients [6]. In Mexico, populations of *B. cockerelli* resistant to carbofuran, malathion, methomyl [6], imidacloprid, [7] and abamectin [8] have already been reported. Therefore, it is necessary to find or generate effective insect management alternatives that do not pollute the environment. In this context, one option for managing *B. cockerelli* is to obtain pest-resistant varieties through genetic improvement. In plants, insect resistance falls into two categories. Antixenosis indicates the presence of morphological or chemical factors of the plant that negatively affect the behavior of the insect, causing the selection of another plant as host, and antibiosis manifests when the plant can negatively affect the growth, development, and reproduction of the insect [9,10,11].

Mexico is considered the center of origin of *C. annuum* [12] and has a great genetic diversity within this species. Therefore, there is a high probability of finding chili pepper varieties resistant to pests and phytopathogens, such as chili ‘Criollo de Morelos’ (CM-334), which is resistant to *Phytophthora capsici* [13], to different phytopathogenic viruses [14,15], and to the nematodes *Meloidogyne incognita*, *M*. *arenaria,* and *M*. *javanica* [16,17]. Previous studies indicating multiple resistance in CM-334 and observations made under field conditions showing that the ‘Criollo de Jojutla’ (CJ-2018) variety is barely colonized by insect pests suggest that both varieties could be resistant to *B. cockerelli.* To verify this, we decided to evaluate the effect of these chili varieties on *B. cockerelli* behavior and biology.

## 2. Materials and Methods

### 2.1. Biological Material

The chili pepper varieties evaluated were: ‘Criollo de Morelos’ (CM-334) and ‘Criollo de Jojutla’ (CJ-2018). A commercial variety susceptible to *B. cockerelli* (‘Arbol’ (CA)) was used as a control. The seeds of each variety were disinfected with 1% sodium hypochlorite for one minute, then rinsed with sterile tap water three times and placed in humid chambers at 26 ± 2 °C for germination. One week after germination, seedlings of each variety were transplanted into pots 6.5 cm in diameter by 5.5 cm in height containing a sterile mixture of peat moss and perlite (2:1). The plants were watered every other day with sterile tap water, fertilized once a week with YaraMila COMPLEX^®^ (NPK, 12-11-18: 3 g/L), and kept in a growth room at 26 ± 2 °C with a photoperiod of 12 h of light for 12 h of darkness, until they had the 6th true leaf, to be used in the experiments. The *Bactericera cockerelli* insects were originally collected from a chili field at Xalatzala town, located in the municipality of Tlapa de Comonfort, Guerrero, Mexico. The colony was established and reproduced under controlled conditions (27± 2 °C, 36% humidity, and a photoperiod of 12 h of light for 12 h of darkness). One week after emergence, adults were used for the tests.

### 2.2. Oviposition Preference

#### 2.2.1. Choice Assays

To evaluate the oviposition preference of *B. cockerelli* over any of the pepper varieties, they were allowed to oviposit for 6 days under laboratory conditions (27 ± 2 °C, 36% humidity, and a photoperiod of 12 h of light for 12 h of darkness). After this time, the adults were removed, and the eggs were counted under a stereoscopic microscope (Stemi DV4, Carl Zeiss, Germany). For this trial, the following was done: one plant of each variety was placed in cages of 40 × 40 × 40 cm, randomly with an equidistant separation. Fifteen pairs of insects were introduced into a 50 mL Falcon™ conical tube, which was placed in the center of the upper part of the cage; the insects were immediately released, and in this way the females could select the variety of pepper to oviposit. This was considered a replica, and 10 replicates were performed. The test was repeated twice.

#### 2.2.2. Non-Choice Assays

To examine the oviposition rate of *B. cockerelli* on the chili varieties, the adults were allowed to oviposit on chili plants for 1, 2, 4, and 6 days under controlled conditions. After each time, the adults were removed, and the eggs were counted under a stereoscopic microscope (Stemi DV4, Carl Zeiss, Germany). For each evaluated time, a trial was placed under a completely randomized design with 10 repetitions per variety. In each trial, the following was done: five pairs of insects were selected and placed inside a 10 × 10 × 25 cm anti-aphid fabric cage. The cage was immediately placed on the chili plant, and to prevent the insects from escaping, the cages were adhered and sealed on the back side of the pots. This procedure was carried out in each repetition of each variety. The tests were repeated twice.

### 2.3. Growth and Survival of Bactericera cockerelli

To determine the development and viability of the immature stages of *B. cockerelli* fed on each pepper variety, the insects were placed as described in the non-choice oviposition tests section. In this test, 10 pairs of insects were used. They were allowed to oviposit for only 24 h. After this time, the insects were removed and only 30 eggs per plant or repetition were left. From that moment on, the development of the insects was monitored every 24 h under a stereoscopic microscope (Stemi DV4, Carl Zeiss, Germany). Each variety had 10 repetitions, and the experiment was repeated twice.

### 2.4. Statistical Analysis

The data obtained from each test repetition were analyzed together because they did not show significant differences. The data were transformed (square root) due to their non-normal distribution, and prior to the one-way analysis of variance (ANOVA), it was verified that they met the statistical assumptions. The comparison of means was performed using the Tukey test (*p* < 0.05). The R program version 4.1.1 [18] was used for the analyses.

## 3. Results

### 3.1. Oviposition Preference, Choice, and Non-Choice Assays for Six Days

In the choice oviposition trial, *B. cockerelli* in CJ-2018 reduced the number of laid eggs by 92.17% compared to the CA control. In contrast, in CM-334 *B. cockerelli* increased the number of laid eggs by 100.55% compared to the control (*F* = 732; df = 2, 18; *p* < 0.001) (Table 1). In the non-choice 6-day oviposition trials, *B. cockerelli* oviposition was reduced on CM-334 by 12.31% and by 80.18% on CJ-2018 compared to the CA control (*F* = 279.3; df = 2, 27; *p* < 0.001) (Table 1). 

### 3.2. Oviposition Preference, Choice, and Non-Choice Assays at Different Times

**Figure 1:**
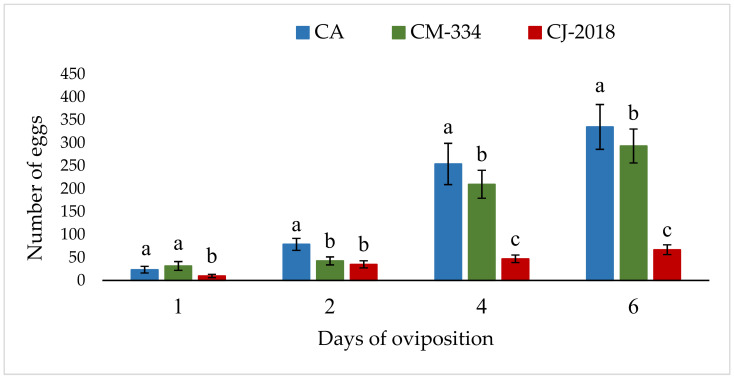
Number of eggs laid by females of *Bactericera cockerelli* in three varieties of pepper, on different days of oviposition. CA: chili pepper ‘Arbol’, CM-334: ‘Criollo de Morelos 334’, CJ-2018: ‘Criollo de Jojutla 2018’. Each point represents the average of 10 repetitions corresponding to two repetitions of the test. Values with different letters each time were significantly different (Tukey test *p* < 0.05).

According to the non-choice test at different times, after one day *B. cockerelli* showed a 58.11% (9.80 ± 3.68) decrease in the oviposition on CJ-2018, and an increase of 35% (31.60 ± 9.47) in the oviposition on CM-334 compared to the CA control (23.40 ± 7.15) (*F* = 30.04; df = 2, 27; *p* < 0.001). After two days, *B. cockerelli* showed a 55.40 (35.10 ± 7.69) and 45.87% (42.60 ± 8.75) decrease in the oviposition on CJ-2018 and CM-334 compared to the CA control (78.70 ± 13.09), respectively (*F* = 49.34; df = 2, 27; *p* < 0.001). After four days, the *B. cockerelli* oviposition was reduced on CJ-2018 by 81.45% (47.10 ± 8.24) and by 17.48% (209.60 ± 30.49) on CM-334 compared to the control (254.00 ± 45.00) (*F* = 207.3; df = 2, 27; *p* < 0.001). This trend was maintained after six days (Section 3.1 and Figure 1).

### 3.3. Development and Survival of Bactericera cockerelli

**Figure 2:**
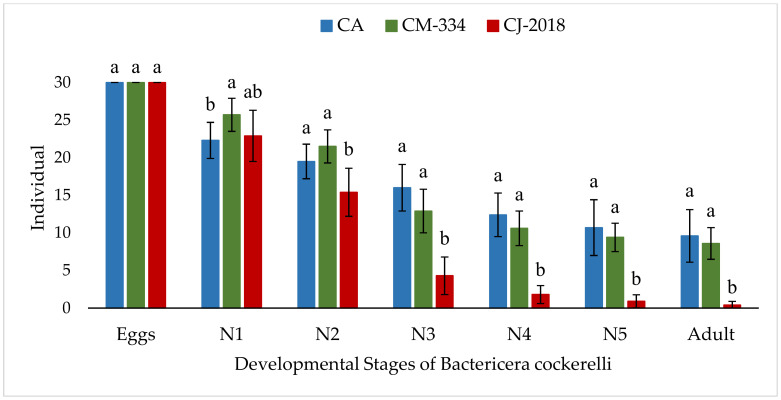
Development of *Bactericera cockerelli* fed with chili plants of the ‘Arbol’ variety (CA), ‘Criollo de Morelos’ (CM-334), or ‘Criollo de Jojutla’ (CJ-2018). N1: First instar nymph, N2: Second instar nymph, N3: Third instar nymph, N4: Fourth instar nymph, N5: Fifth instar nymph. Each point represents the average of 10 repetitions with 30 individuals each. Values with different letters in each developmental stage are significantly different (Tukey test *p* < 0.05).

In the development trial, the first-instar nymphs (N1) hatched from eggs on CM-334 (85.66%; 25.70 ± 2.26) were significantly different, while on CJ-2018 (76.33%; 22.90 ± 3.41) it was similar compared to the control (74.33%; 22.30 ± 2.50) (*F* = 4.29; df = 2, 27; *p* < 0.05) (Figure 2). Subsequently, in CA a gradual decrease in nymphs was observed through the different developmental stages, and finally 32.00% (9.60 ± 3.50) of the eggs managed to reach the adult stage. In CM-334, a greater decrease in nymphs was observed at early instar stages, with a greater difference between stages N2 (71.66%; 21.50 ± 2.22) and N3 (43.00%; 12.90 ± 2.96). However, no significant differences were observed through the instar stages compared to the control, and finally 28.66% (8.60 ± 2.11) of the eggs managed to reach the adult stage (Figure 2). On the contrary, once the *B. cockerelli* eggs hatched on CJ-2018, significantly fewer nymphs survived through the different instar stages compared to the CA control. Thus, only 14.33% (4.30 ± 2.54) of the eggs developed to N3 on CJ-2018 compared to 53.33% (16.00 ± 3.20) on the CA control (*F* = 43.32; df = 2, 27; *p* < 0.001). At the end of the assay, only 1.33% (0.4 ± 0.5) of the eggs managed to reach the adult stage on CJ-2018 compared to 32.00% (9.60 ± 3.50) on the CA control (*F* = 85.47; df = 2, 27; *p* < 0.001) (Figure 2).

Under the conditions evaluated, the number of days required by the insects to develop their nymphal stages on each pepper variety was not statistically different (Table S1).

## 4. Discussion

Non-choice and choice oviposition preference assays for six days showed that *B. cockerelli* had no preference for laying its eggs on CJ-2018, indicating that it possesses antixenosis-based resistance. So far, no pepper varieties resistant to *B. cockerelli* have been reported. Oviposition preference assays are commonly used to identify pest-resistant plants based on antixenosis, for example in soybean (*Glycine max*) against *Bemisia tabaci* (Genn.) (Hemiptera: Aleyrodidae) [19,20], *Chrysodeixis includens* (Walker) (Lepidoptera: Noctuidae) [21], and *Anticarsia gemmatalis* (Hübner) (Lepidoptera: Erebidae) [22], as well as in beans (*Phaseolus vulgaris*) and chili (*Capsicum* spp.) against *B. tabaci* [23,24,25,26]. 

In the non-choice trial, *B. cockerelli* laid similar numbers of eggs on both CM-334 and CA; in contrast, in the choice trial, *B. cockerelli* laid more eggs on CM-334 than CA. A possible explanation is that in the non-choice trial, the insects could not move from one variety to another, whereas in the choice trial, *B. cockerelli* preferred to move and laid its eggs on CM-334. This behavior suggests that CM-334 had an attractive effect on the oviposition preference of *B. cockerelli*, as reported for *Bactrocera dorsalis* (Hendel) (Diptera:Tephritidae) in chili varieties that synthesize the volatile β-ocimene, this compound being associated with the attraction of females to the fruits so that they lay their eggs [27]. A similar effect was observed with the volatile β-phellandrene, which was associated with tomato susceptibility to *B. cockerelli* [28].

On the other hand, CM-334 was interesting to evaluate because it is resistant to *M. incognita*, *M. arenaria,* and *M. javanica*, which is mediated by the Me3/7 locus [16,17]. In tomatoes, it has been reported that varieties resistant to *M. incognita* induce the *Mi-1* gene [29,30], also responsible for resistance to the aphid *Macrosiphum euphorbiae* (Thomas) (Hemiptera: Aphididae) [31,32] and *B. tabaci* [33]. In addition, the *Mi-1* gene was associated with resistance against *B. cockerelli* based on antixenosis and antibiosis in tomatoes [34]. Considering this background, we expected that the Me3/7 locus in chili could have a similar effect to that reported with the *Mi-1* gene in tomatoes, so that CM-334 would be resistant to *B. cockerelli*. However, our results indicated that CM-334 was susceptible to *B. cockerelli*. Therefore, the Me3/7 locus responsible for resistance against nematodes was not able to provide resistance to *B. cockerelli*.

The non-choice test at different times indicated that from the first day, *B. cockerelli* perceived CJ-2018 as a plant that was not suitable for oviposition, since the number of laid eggs was reduced by half compared to the control; this behavior was similar at two days and was more evident at four and six days, confirming that the resistance of CJ-2018 against *B. cockerelli* was based on antixenosis.

Resistance based on antixenosis depends on traits such as morphological characteristics (color, shape, type of leaf surface and thickness, presence or absence of trichomes) and the synthesis of defense-related secondary metabolites [9]. Firdaus et al. (2011) reported that *B. tabaci* preferred to laid its eggs on chili plants with a higher density of non-glandular trichomes and a thin cuticle. In contrast, the varieties that showed resistance had glandular trichomes and thick cuticles [23]. Another study identified that phytol extracted from sweet pepper (*C. annuum* var. *angulosum*) leaves was a highly effective deterrent against *Lyriomyza trifolii* (Burgess) (Diptera: Agromyzidae) oviposition [35]. Additionally, it was reported that the volatile 6-methyl-5-hepten-2-one obtained from the Hybrid Green Belt pepper (*C. annuum*) repelled *Aphis gossypii* (Glover) (Hemiptera: Aphididae) in chili and in wheat [36,37]. In this study, the morphological characteristics were not analyzed, although it is unlikely that the resistance of CJ-2018 based on antixenosis is related to these characteristics since the leaves of CJ-2018 and CM-334 are similar (Figure S1). In addition, the cumulative effect observed in the oviposition test at different times suggests that the presence and accumulation of secondary metabolites with a repellent effect in the leaves of CJ-2018 could be related to said resistance. This is a very important aspect that needs to be studied in detail in future work.

Currently, there are no reports of wild or native varieties of pepper resistant to *B. cockerelli*. In contrast, the wild tomato *Solanum habrochaites* was resistant to *B. tabaci* and *B. cockerelli*. This resistance was probably mediated by the compounds 7-epizingiberene and R-curcumene that repelled *B. tabaci* [38]. However, for *B. cockerelli,* this relationship has not been established, although it was speculated that these compounds were involved in the repellent effect [39,40]. Mayo-Hernandez et al. (2019) suggested that the induction of volatile organic compounds such as β-phellandrene and (+)-4-carene in an unidentified variety of wild tomato could be related to a decrease in oviposition of *B. cockerelli* [41,42]. The comparison of the metabolic profile between a susceptible variety (cv. CastleMart) and a resistant variety (RIL LA3952) of tomato identified different metabolites that could be involved in the defense against *B. cockerelli*. The accumulation of p-coumaric acid was associated with resistance, while the accumulation of 4-hydroxybenzoic acid and rutin were associated with a susceptibility to *B. cockerelli*. In addition, the hormones zeatin and salicylic acid showed relatively high concentrations in the susceptible variety and low concentrations in the resistant variety, suggesting that they did not play a relevant role in the defense against *B. cockerelli*, whereas gibberellic acid and jasmonic acid showed higher levels in the resistant variety compared to the susceptible variety, suggesting a possible role in signaling the defense against the insect [28].

The development test showed that CM-334 did not present resistance based on antibiosis, since the individuals that managed to reach the adult stage were similar compared to the control. In contrast, from N2, the number of individuals that survived in each instar stage was significantly lower on CJ-2018, and only 1.33% of the eggs managed to reach the adult stage, suggesting that CJ-2018 had a high antibiosis-based resistance against *B. cockerelli*. It has been observed that this type of resistance may be due to low nutritional quality in plant tissues or to the rapid accumulation of toxic metabolites for insects. It is likely that CJ-2018, when it detected the presence of *B. cockerelli*, synthesized compounds similar to diterpene glycosides and flavonoids that were associated with the antibiosis of *Capsicum annuum* against thrips *Frankliniella occidentalis* (Pergande) (Thysanoptera: Thripidae) and *Thrips parvispinus* (Karny) (Thysanoptera: Thripidae) [43,44]. In the case of *B. cockerelli*, it was reported that tomato lines that have a high production of acyl sugars showed both types of resistance (antibiosis and antixenosis) against this insect [45]. In our case, we cannot rule out whether the antibiosis-based resistance against *B. cockerelli* observed in CJ-2018 was due to poor nutrient quality, the presence of toxic metabolites for the insect, or a combination of both, which can be analyzed in later work.

Our results indicated that CJ-2018 was resistant to *B. cockerelli* and represented the first report of a Criollo pepper variety with natural resistance to this insect. The identification of CJ-2018 as a pepper variety resistant to *B. cockerelli* represents the first step towards the determination and analysis of the genetic bases that confer this resistance trait. Thereafter, the resistance trait could be transferred to pepper varieties of economic importance through a genetic improvement program in order to develop viable alternatives for *B. cockerelli* management. In contrast, varieties resistant to *B. cockerelli* have been reported in the wild tomato *Solanum habrochaites* [40] and in the different genotypes of *Solanum tuberosum*, [46,47], *S. bulbocastanum* [48], and *S. chacoense* [49]. 

## 5. Conclusions

The chili pepper ‘Criollo de Morelos’, CM-334, was susceptible to *B. cockerelli,* and the chili pepper ‘Criollo de Jojutla’, CJ-2018, was resistant to *B. cockerelli*, which was based on antixenosis and antibiosis.

## Figures and Tables

**Table 1:** Preference of oviposition of *Bactericera cockerelli* for 6 days on three varieties of pepper, under two conditions: choice and non-choice.

Variety of Chili	Number of Eggs	
	Choice	Non-Choice
‘Árbol’	358.60 ± 29.64 b	333.80 ± 46.41 a
‘Criollo de Morelos, CM-334’	718.80 ± 50.72 a	292.10 ± 35.13 b
‘Criollo de Jojutla, CJ-2018’	28.40 ± 7.22 c	67.00 ± 9.97 c

Each value represents the average of 10 repetitions ± the standard error corresponding to two experiments. Values with different letters in each column were significantly different (Tukey test *p* < 0.05).

## Data Availability

The data presented in this study are available on request from the corresponding author.

## Supplementary Materials

The following supporting information can be downloaded at: https://www.mdpi.com/article/10.3390/insects13080742/s1, Figure S1: Aerial view of both varieties evaluated. A) ‘Criollo de Morelos’ (CM-334) and B) ‘Criollo de Jojutla’ (CJ.2018). Plants were grown under greenhouse conditions; Table S1: Developmental times of *B. cockerelli* immature stages fed on different chili pepper varieties under controlled conditions.

## References

[1] Servicio de Información Agroalimentaria y Pesquera (SIAP) Panorama Agroalimentario 2021. Conectando Conocimiento Ancentral y Moderno Para Lograr La Autosuficiencia Agroalimentaria. https://nube.siap.gob.mx/gobmx_publicaciones_siap/pag/2021/Panorama-Agroalimentario-2021.

[2] Hansen A.K., Trumble J.T., Stouthamer R., Paine T.D. (2008). A New Huanglongbing Species, “*Candidatus* Liberibacter Psyllaurous”, Found to Infect Tomato and Potato, Is Vectored by the Psyllid Bactericera Cockerelli (Sulc). Appl. Environ. Microbiol..

[3] Liefting L.W., Perez-Egusquiza Z.C., Clover G.R.G., Anderson J.A.D. (2008). A New “*Candidatus* Liberibacter” Species in Solanum Tuberosum in New Zealand. Plant Dis..

[4] Camacho-Tapia M., Rojas-Martínez R.I., Zavaleta-Mejía E., Hernández-Deheza M.G., Carrillo-Salazar J.A., And R.-A.A., Ochoa-Martínez D.L. (2011). Aetiology of Chili Pepper Variegation from Yurécuaro, México. J. Plant Pathol..

[5] Velásquez-Valle R., Reveles-Torres L.R., Mena-Covarrubias J., Salas-Muñoz S., Mauricio-Castillo J.A. (2014). Outbreak of *Candidatus* Liberibacter Solanacearum in Dried Chile Pepper in Durango, Mexico. Agrofaz.

[6] Dávila Medina M.D., Cerna Chávez E., Aguirre Uribe L.A., García Martínez O., Ochoa Fuentes Y.M., Gallegos Morales G., Landeros Flores J. (2012). Susceptibilidad y Mecanismos de Resistencia a Insecticidas En Bactericera Cockerelli (Sulc.) En Coahuila, México. Rev. Mex. Cienc. Agríc..

[7] Cerna E., Ochoa Y., Aguirre L.A., Flores M., Landeros J. (2013). Determinación de La Resistencia a Insecticidas En Cuatro Poblaciones Del Psílido de La Papa Bactericera Cockerelli (Sulc) (Hemiptera: Triozidae). Rev. Int. Bot. Exp..

[8] Cerna-Chávez E., Bautista O.H., Flores J.L., Uribe L.A., Fuentes Y.M.O. (2015). Insecticide-Resistance Ratios of Three Populations of Bactericera Cockerelli (Hemiptera: Psylloidea: Triozidae) in Regions of Northern Mexico. Fla. Entomol..

[9] Smith C.M. (2005). Plant Resistance to Arthropods.

[10] Koch K.G., Chapman K., Louis J., Heng-Moss T., Sarath G. (2016). Plant Tolerance: A Unique Approach to Control Hemipteran Pests. Front. Plant Sci..

[11] Mitchell C., Brennan R.M., Graham J., Karley A.J. (2016). Plant Defense against Herbivorous Pests: Exploiting Resistance and Tolerance Traits for Sustainable Crop Protection. Front. Plant Sci..

[12] Kraft K.H., Brown C.H., Nabhan G.P., Luedeling E., De Jesús Luna Ruiz J., D’Eeckenbrugge G.C., Hijmans R.J., Gepts P. (2014). Multiple Lines of Evidence for the Origin of Domesticated Chili Pepper, Capsicum Annuum, in Mexico. Proc. Natl. Acad. Sci. USA.

[13] Villar-Luna E., Rojas-Martínez R.I., Reyes-Trejo B., Gómez-Rodríguez O., Zavaleta-Mejía E. (2017). Mevalonate Pathway Genes Expressed in Chilli CM334 Inoculated with Phytophthora Capsici and Infected by Nacobbus Aberrans and Meloidogyne Enterolobii. Eur. J. Plant Pathol..

[14] Boiteux L.S., Cupertino F.P., Silva C., Dusi A.N., Monte-Neshich D.C., Van Der Vlugt R.A., Fonseca M.E. (1996). Resistance to Potato Virus Y (Pathotype 1–2) in Capsicum Annuum and Capsicum Chinense Is Controlled by Two Independent Major Genes. Euphytica.

[15] Janzac B., Fabre M.F., Palloix A., Moury B. (2009). Phenotype and Spectrum of Action of the Pvr4 Resistance in Pepper against Potyviruses, and Selection for Virulent Variants. Plant Pathol..

[16] Djian-Caporalino C., Pijarowski L., Januel A., Lefebvre V., Daubèze A., Palloix A., Dalmasso A., Abad P. (1999). Spectrum of Resistance to Root-Knot Nematodes and Inheritance of Heat-Stable Resistance in in Pepper (*Capsicum annuum* L.). Theor. Appl. Genet..

[17] Djian-Caporalino C., Fazari A., Arguel M.J., Vernie T., Vande Casteele C., Faure I., Brunoud G., Pijarowski L., Palloix A., Lefebvre V. (2007). Root-Knot Nematode (*Meloidogyne* spp.) Me Resistance Genes in Pepper (*Capsicum annuum* L.) Are Clustered on the P9 Chromosome. Theor. Appl. Genet..

[18] R Core Team (2021). R: A Language and Environment for Statistical Computing.

[19] do Valle G.E., Lourenção A.L., Pinheiro J.B. (2012). Adult Attractiveness and Oviposition Preference of Bemisia Tabaci Biotype B in Soybean Genotypes with Different Trichome Density. J. Pest Sci..

[20] Baldin E.L.L., Cruz P.L., Morando R., Silva I.F., Bentivenha J.P.F., Tozin L.R.S., Rodrigues T.M. (2017). Characterization of Antixenosis in Soybean Genotypes to Bemisia Tabaci (Hemiptera: Aleyrodidae) Biotype B. J. Econ. Entomol..

[21] Schlick-Souza E.C., Baldin E.L.L., Morando R., Lourenção A.L. (2018). Antixenosis to Chrysodeixis Includens (Lepidoptera: Noctuidae) among Soybean Genotypes. Bragantia.

[22] Ongaratto S., Silveira C.M., Santos M.C., Gorri J.E.R., Sartori M.M.P., Hunt T.E., Lourenção A.L., Baldin E.L.L. (2021). Resistance of Soybean Genotypes to Anticarsia Gemmatalis (Lepidoptera: Erebidae): Antixenosis and Antibiosis Characterization. J. Econ. Entomol..

[23] Firdaus S., Van Heusden A., Harpenas A., Supena E.D.J., Visser R.G.F., Vosman B. (2011). Identification of Silverleaf Whitefly Resistance in Pepper. Plant Breed..

[24] Da Silva A.G., Boica Junior A.L., Farias P.R.S., Rodrigues N.E.L., De Souza B.H.S., Bottega D.B., Chiorato A.F. (2014). Non-Preference for Oviposition and Antibiosis in Bean Cultivars to Bemisia Tabaci Biotype B (Hemiptera: Aleyrodidae). Rev. Colomb. Entomol..

[25] Pantoja K.F.C., Rocha K.C.G., Melo A.M.T., Marubayashi J.M., Baldin E.L.L., Bentivenha J.P.F., Gioria R., Kobori R.F., Pavan M.A., Krause-Sakate R. (2018). Identification of Capsicum Accessions Tolerant to Tomato Severe Rugose Virus and Resistant to Bemisia Tabaci Middle East-Asia Minor 1 (MEAM1). Trop. Plant Pathol..

[26] Hernández-Alvarado L.A., Ruiz-Sánchez E., Latournerie-Moreno L., Garruña-Hernández R., González-Mendoza D., Chan-Cupul W. (2019). Resistance of Capsicum Annuum Genotypes to Bemisia Tabaci and Influence of Plant Leaf Traits. Rev. Fitotec. Mex..

[27] Syamsudin T.S., Kirana R., Karjadi A.K., Faizal A. (2022). Characteristics of Chili (*Capsicum Annuum* L.) That Are Resistant and Susceptible to Oriental Fruit Fly (*Bactrocera dorsalis* Hendel) Infestation. Horticulturae.

[28] Lee J.H.J., Awika H.O., Jayaprakasha G.K., Avila C.A., Crosby K.M., Patil B.S. (2020). Tomato Metabolic Changes in Response to Tomato-Potato Psyllid (*Bactericera Cockerelli*) and Its Vectored Pathogen *Candidatus* Liberibacter Solanacearum. Plants.

[29] Chen R., Li H., Zhang L., Zhang J., Xiao J., Ye Z. (2007). CaMi, a Root-Knot Nematode Resistance Gene from Hot Pepper (*Capsium Annuum* L.) Confers Nematode Resistance in Tomato. Plant Cell Rep..

[30] Gilbert J., McGuire D. (1956). Inheritance of Resistance to Severe Root-Knot from Meloidogyne Incognita in Commercial-Type Tomatoes. Proc. Am. Soc. Hortic. Sci..

[31] Rossi M., Goggin F.L., Milligan S.B., Kaloshian I., Ullman D.E., Williamson V.M. (1998). The Nematode Resistance Gene Mi of Tomato Confers Resistance against the Potato Aphid. Proc. Natl. Acad. Sci. USA.

[32] Vos P., Simons G., Jesse T., Wijbrandi J., Heinen L., Hogers R., Frijters A., Groenendijk J., Diergaarde P., Reijans M. (1998). The Tomato Mi-1 Gene Confers Resistance to Both Root-Knot Nematodes and Potato Aphids. Nat. Biotechnol..

[33] Nombela G., Williamson V.M., Muñiz M. (2003). The Root-Knot Nematode Resistance Gene Mi-1.2 of Tomato Is Responsible for Resistance against the Whitefly *Bemisia Tabaci*. Mol. Plant-Microbe Interact..

[34] Casteel C.L., Walling L.L., Paine T.D. (2006). Behavior and Biology of the Tomato Psyllid, Bactericerca Cockerelli, in Response to the Mi-1.2 Gene. Entomol. Exp. Appl..

[35] Kashiwagi T., Mikagi E., Mekuria D.B., Borua A.D., Tebayashi S.I., Kim C.S. (2005). Ovipositional Deterrent on Mature Stage of Sweet Pepper, Capsicum Annuum, against Liriomyza Trifolii (Burgess). Z. Naturforsch. C J. Biosci..

[36] Quiroz A., Pettersson J., Pickett J.A., Wadhams L.J., Niemeyer H.M. (1997). Semiochemicals Mediating Spacing Behavior of Bird Cherry-Oat Aphid. J. Chem. Ecol..

[37] da Costa J.G., Pires E.V., Riffel A., Birkett M.A., Bleicher E., Sant’Ana A.E.G. (2011). Differential Preference of Capsicum Spp. Cultivars by Aphis Gossypii Is Conferred by Variation in Volatile Semiochemistry. Euphytica.

[38] Bleeker P.M., Diergaarde P.J., Ament K., Guerra J., Weidner M., Schütz S., de Both M.T.J., Haring M.A., Schuurink R.C. (2009). The Role of Specific Tomato Volatiles in Tomato-Whitefly Interaction. Plant Physiol..

[39] Levy J., Tamborindeguy C. (2014). Solanum Habrochaites, a Potential Source of Resistance against *Bactericera Cockerelli* (Hemiptera: Triozidae) and “*Candidatus* Liberibacter Solanacearum”. J. Econ. Entomol..

[40] Avila C.A., Marconi T.G., Viloria Z., Kurpis J., Del Rio S.Y. (2019). *Bactericera Cockerelli* Resistance in the Wild Tomato *Solanum Habrochaites* Is Polygenic and Influenced by the Presence of *Candidatus* Liberibacter Solanacearum. Sci. Rep..

[41] Mayo-Hernández J., Flores-Olivas A., Valenzuela-Soto J., Rodríguez-Pagaza Y., Vega-Chávez J., Hernández-Castillo F., Aguirre-Uribe L. (2018). Bactericera Cockerelli Sulc Oviposition Preference and Development on Three Tomato Varieties. Southwest. Entomol..

[42] Mayo-Hernández J., Ramírez-Chávez E., Molina-Torres J., Guillén-Cisneros M.D.L., Rodríguez-Herrera R., Hernández-Castillo F., Flores-Olivas A., Valenzuela-Soto J.H. (2019). Effects of *Bactericera cockerelli* Herbivory on Volatile Emissions of Three Varieties of *Solanum lycopersicum*. Plants.

[43] Maharijaya A., Vosman B., Steenhuis-Broers G., Harpenas A., Purwito A., Visser R.G.F., Voorrips R.E. (2011). Screening of Pepper Accessions for Resistance against Two Thrips Species (*Frankliniella occidentalis* and *Thrips parvispinus*). Euphytica.

[44] Maharijaya A., Vosman B., Pelgrom K., Wahyuni Y., de Vos R.C.H., Voorrips R.E. (2019). Genetic Variation in Phytochemicals in Leaves of Pepper (*Capsicum*) in Relation to Thrips Resistance. Arthropod-Plant Interact..

[45] Li Z., Kund G., De Jong D.M., Feng X., Mutschler M.A., Trumble J.T. (2019). Effects of High-Level Acylsugar-Producing Tomato Lines on the Development of Tomato Psyllids (Hemiptera: Triozidae). J. Econ. Entomol..

[46] Butler C.D., Gonzalez B., Manjunath K.L., Lee R.F., Novy R.G., Miller J.C., Trumble J.T. (2011). Behavioral Responses of Adult Potato Psyllid, *Bactericera Cockerelli* (Hemiptera: Triozidae), to Potato Germplasm and Transmission of *Candidatus* Liberibacter Psyllaurous. Crop Prot..

[47] Diaz-Montano J., Vindiola B.G., Drew N., Novy R.G., Miller J.C., Trumble J.T. (2014). Resistance of Selected Potato Genotypes to the Potato Psyllid (Hemiptera: Triozidae). Am. J. Potato Res..

[48] Cooper W.R., Bamberg J.B. (2014). Variation in Bactericera Cockerelli (Hemiptera: Triozidae) Oviposition, Survival, and Development on Solanum Bulbocastanum Germplasm. Am. J. Potato Res..

[49] Fife A.N., Cruzado K., Rashed A., Novy R.G., Wenninger E.J. (2020). Potato Psyllid (Hemiptera: Triozidae) Behavior on Three Potato Genotypes with Tolerance to “*Candidatus* Liberibacter Solanacearum”. J. Insect Sci..

