# Supplementary material for: Chili Pepper Jojutla Morelos (Capsicum annuum L.), CJ-2018: A Variety Resistant to Bactericera cockerelli
Source: Insects. 2022 Aug 18;13(8):742. doi: 10.3390/insects13080742 (PMC9409313; doi:10.3390/insects13080742)
Supplement: Supplementary file 1 [file insects-13-00742-s001.zip › insects-1806685-supplementary.pdf]

Supplementary Materials

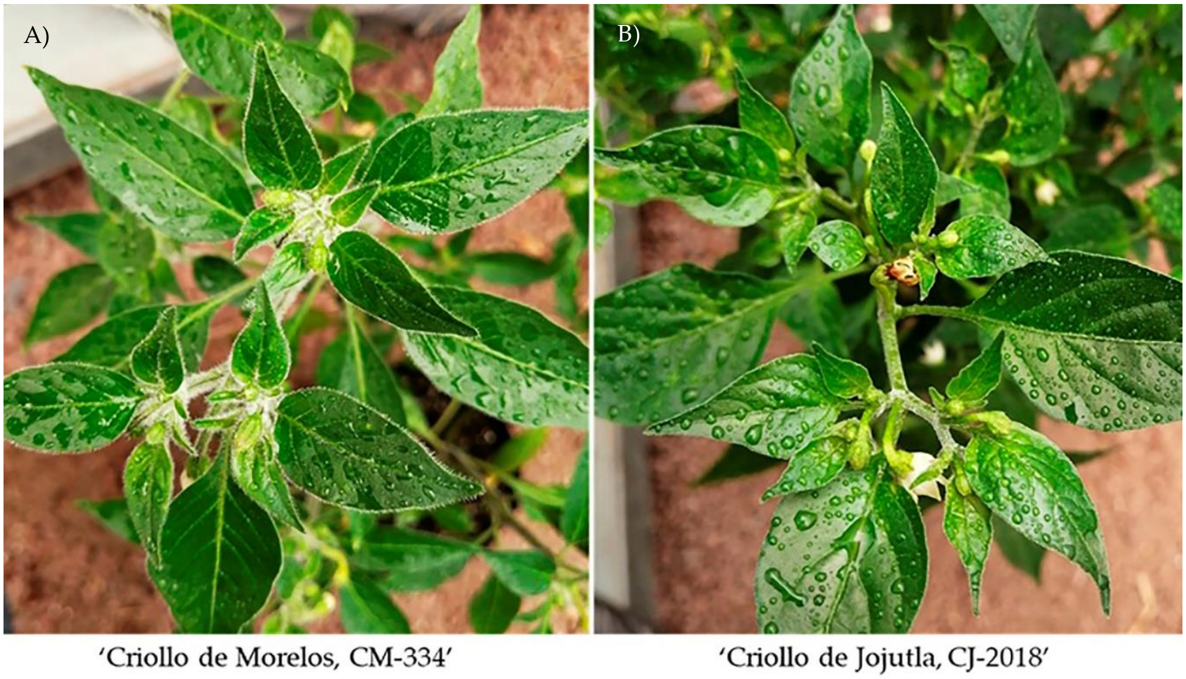

Figure S1. Aerial view of both varieties evaluated. A) ´Criollo de Morelos´ (CM-334) and B) ´Criollo de Jojutla´ (CJ.2018). Plants were grown under greenhouse conditions.

Table S1. Developmental times of *B. cockerelli* immature stages fed on different chili pepper varieties under controlled conditions.

Chili pepper variety	Developmental time in days					
	Eggs	First instar Nymph	Second instar Nymph	Third instar Nymph	Fourth instar Nymph	Fifth instar Nymph
Árbol	5.90 ± 0.54[a]	4.65 ± 0.58 [a]	3.62 ± 0.58 [a]	3.58 ± 0.58 [a]	3.59 ± 0.58 [a]	4.61 ± 0.59 [a]
Criollo de Morelos (CM-334)	5.77 ± 0.61[a]	4.62 ± 0.59 [a]	3.55 ± 0.62 [a]	3.47 ± 0.60 [a]	3.40 ± 0.55 [a]	4.39 ± 0.53 [a]
Criollo de Jojutla (CJ-2018)	6.45 ± 0.50[a]	4.83 ± 0.52 [a]	4.02 ± 0.67 [a]	4.27 ± 0.66 [a]	4.33 ± 0.70 [a]	----

Each point represents the average of 10 repetitions corresponding to two repetitions of the test. Values with different superscript letters in each column are significantly different (Tukey test $p < 0.05$). During the experiment only one egg managed to reach the adult stage on CJ2018. It took 6 days to develop from Fifth instar nymph to adult.
